# ‘So it’s real’: Fibromyalgia, diagnostic injustice, and the case for a shared system response

**DOI:** 10.1016/j.clinme.2026.100646

**Published:** 2026-09-17

**Authors:** Surabhi Wig

**Affiliations:** a Department of Rheumatology, Bolton NHS Trust, Bolton BL35BN, United Kingdom; b University of Greater Manchester, United Kingdom

**Keywords:** Fibromyalgia, Diagnostic delay, Nociplastic pain, Central sensitisation, Health inequalities, Primary care

## Abstract

Fibromyalgia affects 2–4.5% of the UK population, yet remains among the most poorly recognised and inequitably managed conditions in primary and secondary care. Diagnostic delay averages six years with direct clinical and economic consequences, compounded by persistent stigma rooted in historical scepticism of patients’ symptomatology. Recent guidance from the RCP, BSR, GIRFT, and NICE has sought to improve diagnostic clarity and management pathways, yet implementation remains inconsistent and fragmented across England. Current referral arrangements and service provision create institutional barriers that fall disproportionately on patients in deprived areas. This paper examines these systemic failures and argues for a coordinated, multi-level response spanning diagnostic confidence in primary care, specialist consultation as a clinical intervention, equitable access to pain services, and workforce training reform. The goal is not specialist ownership, but integrated contribution from every part of the healthcare pathway.

Recently, I gave two patients a fibromyalgia diagnosis in the same clinic. The first was a middle aged woman who had been symptomatic for nearly a decade. With tears rolling down her face, she said, ‘So it’s real. I’m so relieved’. The second stood up before I finished speaking and said he would be seeking a second opinion. Both reactions are entirely rational. They are what happens when patients absorb years of medical ambivalence and then receive a diagnosis the profession has treated as suspect. Fibromyalgia is among the most common conditions seen across primary and secondary care, yet the clinical literature largely leaves untouched why patients feel ashamed of this diagnosis, why so many reject it, and what medicine as a whole can now do about it.

## The burden of delayed diagnosis

Fibromyalgia is not rare. NHS estimates place UK prevalence at 2–4.5% of the population^1^, up to three million people, with incidence approximately 40% higher in deprived areas^2^. Mean time to diagnosis exceeds six years, and delay directly worsens outcomes^3^. The cost of that delay is not only clinical. A UK primary care study demonstrated that failure to diagnose fibromyalgia generates substantial excess NHS expenditure through repeated GP consultations, investigations, and prescriptions; making the diagnosis was associated with a reduction in resource use of approximately £66 per patient per six-month period^4^. A broader systematic review confirms that fibromyalgia imposes an economic burden comparable to diabetes and hypertension, yet without equivalent recognition or resource allocation^5^.

In postindustrial towns like Bolton, where I work, the condition is more common and the pathway to diagnosis longer. These statistics are not marginal, and they are not evenly distributed. The inconsistency between guidance and practice is not primarily a story of medical scepticism; it reflects genuine challenges of resource allocation, time constraints in both primary and secondary care, and competing clinical priorities. Yet it is a story that demands urgent attention precisely because these systemic pressures have consequences that fall hardest on those least able to absorb them.

## The biology is real, but evidence remains incomplete

The most persistent myth surrounding fibromyalgia is that the pain is psychological. It is not. The IASP classifies fibromyalgia under ‘nociplastic pain’: altered nociception without peripheral tissue damage^6^. Fibromyalgia involves central sensitisation and recognised peripheral mechanisms including small fibre neuropathy. It is a disorder of the nervous system, not of the patient’s credibility.

However, the picture is evolving. While nociplastic pain provides a mechanistic framework, emerging research suggests considerable biological heterogeneity within the fibromyalgia phenotype. Recent work has identified dysregulated inflammatory markers in some patients, and growing evidence points to dysimmune mechanisms in subsets of the fibromyalgia population, particularly those with post-viral presentation. Equally, fibromyalgia sits within a broader spectrum of central sensitivity conditions including chronic fatigue syndrome (CFS), post-viral fatigue syndromes, and long-COVID; conditions that share overlapping clinical features but may have distinct underlying pathologies and require tailored management approaches. Current diagnostic criteria capture a clinical syndrome, but future research will likely enable finer stratification based on underlying mechanisms, allowing more targeted therapeutic approaches. This should not delay current diagnosis and support; rather, it underscores why the diagnostic consultation itself must be treated as a therapeutic intervention that validates patients’ experience while leaving room for evolving understanding.

## Stigma as a clinical determinant

Fibromyalgia’s predecessor labels such as fibrositis and psychogenic rheumatism were applied predominantly to women whose pain lacked a structural correlate, implying they were fragile or fabricating. Those attitudes have not fully disappeared. A 2025 metasynthesis identified three dominant themes in the fibromyalgia stigma experience: symptom invisibility, diagnostic delay, and gender stereotyping, with consequences including eroded trust in clinicians and worsened suffering^7^. UK qualitative research confirms this: patients describe feeling dismissed or disbelieved, and invalidating language compounds the psychological burden of the diagnosis^8^. The stigma is not incidental to the condition; it is part of its clinical course.

The problem extends beyond patients’ perceptions. A 2024 systematic review and meta-analysis found that while most physicians endorsed fibromyalgia as a distinct clinical entity, half considered it primarily a psychosocial condition and knowledge of formal diagnostic criteria was more likely among rheumatologists (69%) than GPs (38%)^9^. A further systematic review documented that healthcare professionals across specialties hold variable attitudes and may view fibromyalgia patients as difficult, attributing this in part to inadequate training rather than clinical reality^10^. These are not character flaws but structural explanations for why diagnostic delay persists despite years of guideline development, and they implicate the whole system.

## A fractured guidance architecture and its consequences

It is against this backdrop that the UK’s recent guidance architecture must be judged. The 2022 RCP diagnostic guideline provides a structured framework for earlier identification and states that a well-conducted diagnostic consultation can itself be therapeutic^11^. It recommends secondary care referral where diagnostic uncertainty exists, supported by a 2024 BSR audit finding that a significant proportion of patients referred with suspected fibromyalgia had alternative or additional diagnoses^12^.

Against this, both the GIRFT report (2021) and the BSR referral criteria (2021) direct that suspected fibromyalgia should be managed in primary care, with the BSR permitting referral only where diagnostic uncertainty is explicitly identified, a threshold that functions as a near-blanket restriction when GP diagnostic confidence is this variable. The PACFiND study found that three in ten GPs reported not diagnosing fibromyalgia, driven by low diagnostic confidence^13^. The resulting situation is not one where primary care is empowered to manage fibromyalgia; it is one where patients with diagnostic uncertainty are neither investigated in secondary care nor confidently diagnosed in primary care, leaving them in limbo.

Redirecting to primary care without addressing diagnostic confidence is displacement, not solution. NICE guideline NG193 represents a paradigm shift towards shared understanding of pain mechanisms, exercise, and psychological therapies^14^. The implementation challenge is acute: those services are absent or severely limited in precisely the areas where fibromyalgia prevalence is highest. A policy that works in Surrey fails in Salford.

## Towards a system response: what each part must do

Fibromyalgia is not a rheumatological condition in the way that vasculitis or myositis are – it does not require ongoing specialist management for most patients. Long-term care sits appropriately in primary care, supported by community services, allied health professionals, and for refractory or complex cases, multidisciplinary pain clinics. But that clarity of destination does not resolve the current failure: no part of the system currently manages this condition consistently well, and institutional fragmentation means patients are shuttled between parts of the system that view responsibility as belonging elsewhere.

## Secondary care: the diagnostic consultation as intervention

For secondary care, the diagnostic consultation must be treated as a clinical intervention, not an administrative endpoint^11^. A specialist who explains central sensitisation clearly, uses language the patient can carry with them, and acknowledges what they have endured can undo years of harmful narrative in a single appointment. The RCP guideline already states this. A structured one-stop model combining diagnostic confirmation, education about pain mechanisms, and a written care plan returned to the GP is time-limited, resists dependency, and gives primary care something to build on rather than a closed door. Examples from centres that have implemented this model, such as dedicated fibromyalgia consultation pathways that combine rheumatology input with structured pain education and reduce onwards referral, demonstrate that this approach is both feasible and valued by patients. The burden of diagnosis should not fall solely on either setting; secondary care can provide clarity where genuine uncertainty exists, after which the patient returns, better supported, to a primary care team equipped to manage them.

## Access to pain services

For patients with refractory or complex presentations, multidisciplinary pain clinics offer an evidence-based option that neither primary nor secondary care is well placed to replicate alone. NICE NG193 recommends their use^14^, yet access is strikingly uneven; pain services are thinnest in precisely the areas of highest fibromyalgia burden. EULAR guidelines similarly recommend a stepped, individualised approach incorporating aerobic exercise, psychological therapies, and patient education^15^. These are evidenced interventions that remain largely undelivered in deprived areas. Some regions have begun to address this through integrated community pain services that combine physiotherapy, cognitive behavioural therapy, and peer support in accessible primary care settings, improving patient satisfaction and reducing secondary care referrals. Expanding such models requires commissioners, rheumatology, and primary care to make the case collectively. The institutional authority to do so exists across the system, if it is used.

## Workforce training

Training must change across the whole workforce. The evidence that most clinicians lack adequate knowledge of fibromyalgia reflects a failure of undergraduate and postgraduate curricula that affects GPs, physicians, allied health professionals, and specialists alike^9^, ^10^. Qualitative research with medical students has shown that fibromyalgia receives little formal curriculum teaching, with students’ attitudes shaped instead by the ‘hidden curriculum’, absorbing the scepticism of clinical teachers rather than evidence-based understanding^16^. Structured exposure to fibromyalgia consultations and the communication skills required to deliver a diagnosis well should sit in training programmes across disciplines. Combined with data on the downstream harm of diagnostic delay^3^, ^7^, this is a patient safety issue.

Empowering GPs to diagnose fibromyalgia confidently is as important as any secondary care intervention. The BSR and GIRFT have already developed structured advice and guidance templates for fibromyalgia, and these deserve active promotion and training implementation rather than passive availability. A GP who can make this diagnosis well, explain it clearly, and initiate a management plan does not need to refer. Building that confidence in primary care is more scalable and more sustainable than any model concentrating expertise in secondary care.

## Reforming referral pathways

Underpinning all of this is a referral framework that needs reform. The current inconsistency between the RCP, BSR, and GIRFT positions reflects a disagreement about resource and responsibility, not about evidence. Patients should not bear the cost of that institutional tension. The BSR criteria should be revised to permit secondary care involvement where diagnostic uncertainty genuinely exists, not as specialty protectionism, but as recognition that the current arrangement is failing people and that investment in diagnostic clarity has downstream benefits.

None of this implies that fibromyalgia belongs to any single specialty. A condition affecting up to three million people cannot be managed well by one part of the system alone. The question is whether each part, primary care, secondary care, pain services, commissioners, and educators, is prepared to play its part in a coordinated response.

## Conclusion

The patient who said ‘so it’s real’ after a decade of symptoms received something she should have had years earlier: validation. The man who walked out may yet find a clinician, in any setting, who explains that his pain is neither imagined nor beneath medicine’s attention. Their different reactions carry the same word: real. What they experienced was real pain, a real diagnosis, and a real institutional failure. Correcting that failure is not a matter of one specialty acting alone, but a system deciding it will. That decision is overdue.

## CRediT authorship contribution statement

**Surabhi Wig:** Writing – review & editing, Writing – original draft, Conceptualization.

## Declaration of generative AI and AI-assisted technologies in the writing process

During the preparation of this work the author used Claude AI for referencing, formatting, and revision. After using this tool, the author reviewed and edited the content as needed and takes full responsibility for the content of the published article.

## Funding

This article did not receive any specific grant from funding agencies in the public, commercial, or not-for-profit sectors.

## Declaration of competing interest

The authors declare that they have no known competing financial interests or personal relationships that could have appeared to influence the work reported in this paper.
